# Alkalizing Properties of Six Calcium-Silicate Endodontic Biomaterials

**DOI:** 10.3390/ma15186482

**Published:** 2022-09-19

**Authors:** Katarzyna Kot, Łukasz Kucharski, Ewa Marek, Krzysztof Safranow, Mariusz Lipski

**Affiliations:** 1 Department of Preclinical Conservative Dentistry and Preclinical Endodontics, Pomeranian Medical University of Szczecin, 70-111 Szczecin, Poland; 2 Department of Cosmetic and Pharmaceutical Chemistry, Pomeranian Medical University of Szczecin, 70-111 Szczecin, Poland; 3 Department of Biochemistry and Medical Chemistry, Pomeranian Medical University of Szczecin, 70-111 Szczecin, Poland

**Keywords:** alkalizing properties, biomaterials, calcium silicate-based cements, pH

## Abstract

Introduction: Calcium silicate-based cements (CSC), are self-setting hydraulic biomaterials widely used for reparative procedures in dentistry and endodontics. These materials possess physical properties, such as ion release, porosity, solubility, and radiopacity. Their biological properties are connected to their alkalizing activity and calcium release capacity. Materials and Methods: Six calcium silicate-based materials were selected for this study: TheraCal LC (Bisco Inc., Schaumburg, IL, USA), MTA Plus (PrevestDenpro, Jammu, India Avalon Biomed Inc., Bradenton, FL, USA), Biodentine (Septodont, Saint-Maur-des-Fossés, France), RetroMTA (BioMTA, Seoul, Korea), MTA Flow (Ultradent Products, Inc., South Jordan, UT, USA), and OrthoMTA (BioMTA, Seoul, Korea). The pH was analyzed immediately after immersion (baseline) and after 1 h, 3 h, 1 day, 2 days, 3 days, 1 week, 2 weeks, 3 weeks, and 1 year with a pH meter, previously calibrated with solutions of known pH. All testing materials had alkaline pH. Results: Analysis of the tested materials showed statistically significant differences in terms of pH changes as a function of the time showed a gradual rise in the pH of all materials. Conclusions: All tested materials exhibited continuous hydroxyl ion release resulting in a rise in pH until the end of time of experience.

## 1. Introduction

Calcium silicate-based cements (CSC), also known as MTA-like cements, are self-setting hydraulic biomaterials widely used for reparative procedures in dentistry and endodontics [1,2,3]. New MTA-like materials based on the original formulation and/or with minor modifications have been introduced in routine clinical practice. Powder of these materials is composed mainly of di- and tri-calcium silicates, calcium phosphate, calcium hydroxide, as well as bismuth oxide or zirconium oxide as radiopacifier [1,2,4,5]. After mixing the powder with water, a chemical reaction occurs and is known as hydration [6,7], which produces primarily calcium hydroxide and forms a sticky calcium silicate hydrate gel (C-S-H gel) [6,8,9]. The indications and clinical applications for calcium silicate-based cements are pulp capping, pulpotomy, apexogenesis, apexification, perforation and resorption repair, root canal sealers, and retrograde surgical filling/root-end sealing [1,2,3,4,5,6,7,8,9,10]. Calcium silicate-based materials possess bio-properties such as biocompatibility and bioactivity (apatite-forming ability in contact with body fluids) [1,2,11]. These materials also have physical properties suitable for their clinical use, such as good sealing [12,13], dimensional changes and solubility in contact with body fluids that are in accordance with ISO standards [14,15,16], and flowability [17,18]. According to manufacturers, calcium silicate-base materials also show alkaline pH. The high pH creates an unfavorable environment for any remaining microorganisms that might survive after cavity preparation or root canal treatment and be the cause of induction or maintain periapical inflammation [11,14,15,16,17,18,19]. Moreover, alkaline pH accelerates apatite nucleation and stimulates the release of alkaline phosphatase and bone morphogenetic protein-2, which participate in the mineralization process [14,15,20]. Therefore, new calcium silicate MTA-like cements have been lately introduced.

TheraCal LC (Bisco Inc., Schaumburg, IL, USA) is a new light-cured, resin-modified calcium silicate cement and is classified as the fourth generation of calcium silicate material (hybrid CSC) [7]. The manufacturer recommends using TheraCal LC as direct and indirect pulp capping material with an ability to stimulate apatite-like precipitates and dentinal bridge and as a restorative liner and base [4,7,11,21]. It also shows physiochemical bonding to dentin, good sealing abilities, and it is well-tolerated by immortalized odontoblast-like cells [21]. MTA Plus (Avalon Biomed Inc., Bradenton, FL, USA) is a powder-liquid system consisting of extremely fine, inorganic powder, which sets with water or an anti-washout gel. It is proposed for treating dental pulp (pulp capping liner, pulpotomy) and root canals (root-end filling, perforation repair, root resorption, apexification, pulpectomy). Different setting times and physical-rheologic properties can be obtained using the gel and varying the powder to gel ratio [7,22,23,24,25]. MTA Plus represents a lower-cost, bioactive calcium silicate-based material as a convenient alternative to the conventional calcium silicate MTA-like cements [22]. MTA Plus presents no cytotoxicity, increases mineralization processes in vitro, and induces the expression of osteogenic markers [24].

Biodentine (Septodont, Saint Maur-des-Fossés, France) is a modified MTA-like material that was introduced to overcome the drawbacks presented by MTA. Biodentine is a fast-setting calcium silicate-based cement used as a dentine substitute, a pulp-capping material, and an endodontic repair material because of its good sealing ability, high compressive strengths, short setting time [26,27] biocompatibility, bioactivity, and biomineralization properties [28].

RetroMTA (BioMTA, Seoul, Korea) material is a mixture of hydrophilic powders that do not contain Portland cement. RetroMTA is a hydraulic bioceramic material formulated for application in perforation and root resorption repair, apical surgery, and vital pulp therapy. It is a powder consisting of fine, hydrophilic particles that set in the presence of water. However, unlike MTA, this material does not contain Portland cement. RetroMTA has a fast setting time (initial time of setting 150 s), good handling properties, no cell toxicity, setting reaction initiated by moisture, no heavy metals, and more excellent washout resistance [29,30,31].

MTA Flow (Ultradent Products, Inc., South Jordan, UT, USA) is a bioactive powder and liquid-gel system consisting of an extremely fine, radiopaque, inorganic powder of tricalcium and dicalcium silicate that sets with a water-based gel [32]. This material, when set, forms a layer of hydroxyapatite, which induces a healing reaction [33]. According to the manufacturer, MTA Flow may be manipulated in different powder/gel ratios, resulting in a smooth consistency and, therefore, is easy to insert into the site indicated. This material’s presentation seems to be innovative compared to the other cements available. MTA Flow has an alkaline pH, low solubility, satisfactory radiopacity, biocompatibility, and induces biomineralization [34,35]. It is proposed in pulp capping, pulpotomy, resorption and perforation repair, apexification, and root-end filling [32,33,34,35].

OrthoMTA (BioMTA, Seoul, Korea) was introduced for apex closure of an immature root, orthograde root canal filling, perforation repair, and retrograde filling. It has a good root canal and dentinal tubule sealing ability, a low expansion rate, and a bioactive characteristic (formation of an interfacial hydroxyapatite layer) [36,37,38]. In the current literature, there are no studies that assess so many commonly used calcium silicate-based materials simultaneously.

The purpose of this in vitro study was to observe time-related changes that occur in the pH values of different calcium silicate-based cements commonly used in the dental practice. The null hypothesis was that there would not be differences between all materials.

## 2. Methods

Six calcium silicate-based materials were selected for this study: TheraCal LC (Bisco Inc., Schaumburg, IL, USA), MTA Plus (Avalon Biomed Inc., Bradenton, FL, USA), Biodentine (Septodont, Saint-Maur-des-Fossés, France), RetroMTA (BioMTA, Seoul, Korea), MTA Flow (Ultradent Products, Inc., South Jordan, UT, USA), and OrthoMTA (BioMTA, Seoul, Korea). Their compositions are outlined in Table 1. Plastic tubes with an internal diameter of 4 mm and a height of 5 mm were weighed before being used for sample preparation. All materials were mixed by the manufacturer’s instructions, except for TheraCal LC, which was packaged in a syringe and light-cure material. Shortly after all materials set, each filled tube was weighed and then placed into a separate dialysis tube containing 10 mL of deionized water (pH 6.8). A negative control group (*n* = 6) consisting of empty tubes was presented to validate the technique used in this study. The vials were sealed and stored in an incubator at 37 °C. A total of 6 samples were used for each material. Adapting the methodology used in other studies allows the results obtained in our research to be compared with other authors. After reviewing the pH evaluation studies of calcium silicate-based materials, the number of samples was calculated. The number of specimen ranged from 5 to 10, but in most publications, it was 6 [6,38,39,40]. Before each measurement, the tubes were shaken for 5 s using triturator Vortex VM-96A (700 rpm, orbital movement with amplitude 4 mm) (Lab Companion, Billerica, MA, USA) to provide uniform hydroxyl ion distribution. The pH was analyzed immediately after immersion (baseline) and after 1 h, 3 h, 1 day, 2 days, 3 days, 1 week, 2 weeks, 3 weeks, and 1 year with a pH meter (CP-401 waterproof, Elmetron, Poland), previously calibrated with solutions of known pH (pH = 4.00, =7.00, and =10.00). A total of 288 measurements were made. The experiment was performed in static conditions (without changing the deionized water) by one person in the Department of Cosmetic and Pharmaceutical Chemistry, Pomeranian Medical University of Szczecin.

## 3. Statistical Analysis

These data are reported as the mean ± standard deviation. To analyze the change in pH levels of the materials at each time point, one- and two-way analysis of variance, ANOVA, followed by Tukey’s multiple comparison test were used. The level of significance was set at *p* < 0.05. The normality was evaluated with the Shapiro–Wilk test.

## 4. Results

**Figure 1:**
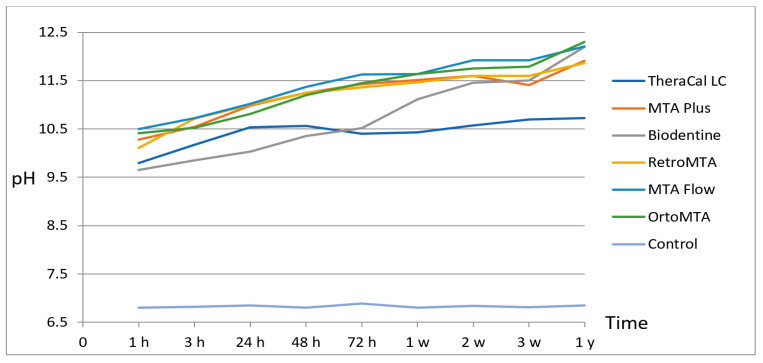
pH changes of materials tested during the course of time of the experiment.

The results of pH changes in all tested materials at different immersion times are presented in Table 2. All testing materials had an alkaline pH (*p* < 0.05). Analysis of the tested materials showed statistically significant differences in pH changes as a function of time showed a gradual rise in the pH of all materials (Figure 1). The pH levels of TheraCal LC varied from 9.79 to 10.72, MTA Plus from 10.28 to 11.91, Biodentine from 9.65 to 12.19, Retro MTA from 10.10 to 11.86, MTA Flow from 10.5 to 12.22, the values for OrthoMTA varied from 10.41 to 12.29. The alkalinity of Biodentine and OrthoMTA was increased gradually by the time of the experiment. In the present study, OrthoMTA showed the highest pH mean values after one year compared to other tested materials. The pH gradually increased in the first 2 days for TheraCal LC and from 1 day to 2 weeks for MTA Plus; subsequently, the pH slowly decreased respectively to materials in the third day and the third week, increasing again. Differences in pH levels between 1 day and 1 year were statistically significant for all tested materials. Significant differences were also observed in different experimental groups at various periods. After 1 h, statistical differences were observed between TheraCal and MTA Flow, control group, and between Biodentine and MTA Flow, OrthoMTA, control group. After 2 weeks, TheraCal showed significant statistical differences with all materials. After 24 h, there were no statistical differences between TheraCal and other testing materials, except for the control group. After one year, differences were noted between TheraCal and all tested materials. The pH of the control group remained unchanged throughout the observation period and was 6.8.

## 5. Discussion

Calcium silicate-based materials have similar basic components and biological properties; however, they differ from each other (setting time, physicochemical properties). MTA-like cements are known to have a high pH resulting from the hydration process [21]. In water, calcium silicates undergo hydrolysis, producing calcium hydroxide and calcium silicate hydrate, which reacts in the presence of physiological fluids producing hydroxyapatite mostly at the surface of the tricalcium silicate paste [41]. High pH (12.5), the hydrated cements contribute to the presence of calcium hydroxide [6]. The bioactivity of calcium silicate-based materials is associated with their ability to release hydroxyl and calcium ions [39]. The release of alkaline phosphatase and bone morphogenetic protein 2 (BMP-2), which are necessary for the mineralization process, is stimulated by the release hydroxide ions [2,34]. Several studies confirmed that calcium silicate-based materials activate hard tissue to repair [2,3,42]. The released hydroxyl ions during the hydration reaction turn the pH environment into alkaline, which inhibits the proliferation of bacteria [4,21,39]. The nature of the mineral particles and cement network structure determinate ion release [8,22]. In the present study, the alkalinizing properties of TheraCal LC (Bisco Inc., Schaumburg, IL, USA), MTA Plus (Avalon Biomed Inc., Bradenton, FL), Biodentine (Septodont, Saint-Maur-des-Fossés, France), RetroMTA (BioMTA, Seoul, Korea), MTA Flow (Ultradent Products, Inc., South Jordan, UT, USA), and OrthoMTA (BioMTA, Seoul, Korea) were evaluated. These materials are commonly used in endodontic treatment as pulp capping, pulpotomy, apexogenesis, apexification, perforation repair, and retrograde filling [1,2,3,4,5,6,7,8,9,10].

In the recent study, time intervals selected for testing were based on the setting times of the materials and previous studies [21,38,43,44]. The pH was analyzed immediately after immersion (baseline) and after 1 h, 3 h, 1 day, 2 days, 3 days, 1 week, 2 weeks, 3 weeks, and 1 year. A comparison of the results of our study with those obtained by other authors may be difficult due to methodological differences. In this in vitro study, deionized water at pH lower than 7 (6.5–6.9) was used as a testing medium, in agreement with other authors [22,31,43,44], to standardize the test conditions and allow a comparison of the data with other studies. However, many authors did not provide the initial pH value of solutions in which samples were immersed [21,40,41,42,43,44,45,46]. In the present study, samples were incubated in the same solution for the entire test period without changing it for the new one after the measurement. After the last measurement (3 weeks), the vials were sealed, stored in an incubator at 37 °C, and untouched, which allowed us to minimize the influence of external factors on the pH measurement. Although, many authors after recording the pH, placed the samples in a fresh solution [22,38,45]. Those authors claimed that a regular exchange of the water where the tested samples were immersed in the experiment was performed to avoid saturation of the medium since it would not present ion exchange, as it occurs in the clinical situation. This is the limitation of our study. To assess to what extent storage medium exchange may affect pH values, whether or not it has an effect, and to what time on saturation, a new investigation is required. The differences in methodology also apply to the size and the volume of the medium in which the samples were stored. In our study, the materials were placed in plastic material with a height of 5 mm and an inner diameter of 4 mm. Thus, the samples were of the same volume and had an identical contact surface with the medium. Other authors placed materials in polyethylene, Teflon, or plastic tubes of lengths 3 mm, 2 mm, 10 mm, 1.6 mm, 5 mm, and diameter respectively 1 mm, 10 mm, 1 mm, 8 mm, 5 mm [38,44,45]. In the present study, all samples were immersed in 10 mL; in other studies, the volume varied from 10 mL to 20 mL [38,40,43,44].

Several methods are available for measuring pH, but no universal standards exist. The use of a pH meter increases the accuracy of the results, as well as provides numeric data that can be analyzed. Although another method can be used, such as the pH curves (titration), this has lower precision and can hinder the accurate interpretation of the results [46]. We used the first method in the present study.

TheraCal LC (Bisco Inc., Schaumburg, IL, USA), as a light-curable resin-modified silicate material sets by hydration, does not include water. Moisture is taken up from the environment and its diffusion within the material. According to manufacturer instructions, this material should be placed on moist dentin. In our study, TheraCal LC proved to be a light-cure material able to increase pH for more than 1 year. Gandolfi et al. [21] reported that TheraCal LC was able to alkalize the surrounding medium initially to pH 10.96–9.28 (3 h–3 days) and subsequently to pH 8.32–8.04 (1 week–4 weeks). The results of another study by the same author [47] showed that TheraCal LC, Biodentine, and MTA Plus induced the alkalization of the soaking water that decreased with time but was still present at 28 days. After 3 h, the pH of soaking water was decreased by TheraCal LC to level 9.53. For Biodentine, TheraCal LC, and MTA Plus, the pH value of the soaking water after 3 days was 10–11, and after 14 days, the pH was decreased to level 8–9. Only Biodentine could keep the pH higher than 9 after 28 days of soaking. TheraCal LC showed the most constant pH among all of the materials.

MTA Plus (Avalon Biomed Inc., Bradenton, FL, USA) has a composition similar to the ProRoot MTA but has a finer mineral powder that can be mixed with two different liquids provided (water or a gel) to obtain materials with varying times of setting [23,24,25]. In our study, MTA Plus showed that the pH initially increased for 2 weeks from 10.28 to 11.59, and in the third week, it decreased to 11.41, then it increased, and one year, it reached the value of 11.91. In contrast, Gandolfi et al. [22] compared the alkalizing properties ofDycal, MTA Plus, and ProRoot MTA. They showed that all three materials created alkaline pH after 3 h of soaking, MTA Plus mixed with gel achieved the pH level at 12. Over 28 days, the pH of all materials gradually decreased. After 28 days, the pH was the highest for Dycal (9.8), MTA Plus (8.29), and MTA Plus gel (7.99) and the lowest for ProRoot MTA (7.1). The pH fluctuation of MTA Plus and TheraCal can be related to the construction of materials. TheraCal consists of only 45% wt mineral material (type III Portland cement) and approximately 45% resin; therefore, its solubility and the ability to release ions are lower than MTA Plus. TheraCal is a resin-modified material; it does not use water as a mixing fluid. The process of hydration of TheraCal depends on fluid uptake through the resin matrix from the surrounding environment. This process is incomplete because of the limitation of moisture diffusion into the material [6]. MTA Plus kit consists of a powder and a gel, which provides washout resistance to the setting material. MTA Plus powder is composed of di- and tricalcium silicate and bismuth oxide, which are the main constituent phases of this material. The extremely fine calcium silicate powder and hydrated polymer gel can impact hydration, ion release, porosity, water sorption, and solubility [22].

New calcium silicate-based materials have been developed to overcome the drawbacks presented by MTA. Biodentine (Septodont, Saint Maur-des-Fossés, France) is among these materials that has captivated attention in the last recent years and has been advocated to be used as a dentine restorative material in addition to endodontic indications similar to those of MTA [26,27,28]. In our study, Biodentine showed a gradual increase in pH throughout the observation period, to finally reached a pH level of 12.19. The pH values observed in the present study were higher than those obtained by other authors. Gandolfi et al. [2] showed that Biodentine induced alkalization of the medium that decreased with time but was still present at 4 weeks. It alkalized the medium to 11.65 fora short time (3 h–1 day), then decreased, and after 28 days, the pH was 9.48 [2]. Aksoy et al. [48] demonstrated similar results reported in other investigations [2,47]. They showed that Biodentine had the highest hydroxyl ion rates (pH 9.6) compared to TheraCal LC (pH 8.2) in a 24-h examination, while the pH gradually decreased during the following measurement periods (7 days–28 days). In both materials, after 7 days, pH started to slowly decrease unlit the last measurement day (Biodentine pH 8.37 and TheraCal LC pH 8.06).

RetroMTA (BioMTA, Seoul, Korea) is a hydraulic bioceramic material proposed for use in similar indications as MTA (pulp capping, perforations, root resorption repair, apexification, and apical surgery) [29,30,31]. According to the manufacturer’s product specification, the pH of RetroMTA is initially 12.5 and decreases to 7.8–8 in 4 weeks [49]. In the present study, the pH of RetroMTA was alkaline, varying from 10.10 to 11.86 after one year. The lower pH values observed in our study compared to the manufacturer may be explained by the different methodology of measurement. Sousa et al. [31] examined the release rate of OH^−^ of ProRoot MTA and RetroMTA in 3, 24, 48, 72 h, and 7 days. As a result of this study, there were no significant differences in the pH levels of ProRoot MTA and RetroMTA throughout the periods of the experiment. The pH of RetroMTA varied from 9.93 to 7.9; the pH of both materials tended to decrease over time.

The poor working properties of MTA-like cements result in a paste that is hard to manipulate. Considering the importance of the ideal flowability that endodontic materials should present reduces the difficulty of handling and facilitating insertion [34]. MTA Flow (Ultradent Products, Inc., South Jordan, UT, USA), high plasticity cement was developed to improve these characteristics. Guimarães et al. [32] compared the alkalizing activity of MTA Flow and MTA Angelus. Both cements showed alkalizing activity. The pH level of the soaking water was reduced with time but was still present until the end of the study (MTA Flow 8.5, MTA Angelus 8.7). At 186 h, the pH level of the MTA Flow was significantly reduced in comparison with the initial time intervals. This result is not in accordance with the results of our study, where the pH of MTA Flow in the 3rd h was 10.72, in the 24th hit was 11.2, and after 168 h it was 11.63. The pH of MTA Flow has reached one of the highest values (12.20) after one year.

The OrthoMTA (BioMTA, Seoul, Korea) has been developed mainly for orthograde root canal obturations as well as retrograde fillings and perforation repairs [36,37]. This material also has a bioactive property; it stimulates the apical foramen to release Ca^2+^, which leads to the formation of a hydroxyapatite layer on its surface. The only study evaluating the alkalizing properties of OrthoMTA is Kim et al. [38]. They demonstrated that ProRoot MTA, OrthoMTA, and Endocem MTA inducted the pH values of the storage water, respectively 11.90, 11.42, and 11.33 on day 7. This result did not differ significantly. In our study, the pH level of OrthoMTA increased gradually during all periods, and it was the highest value (12.29) among all recorded values after the one-year observation. The null hypothesis of this study was rejected; significant differences in pH changes among the calcium silicate-based materials tasted have been shown.

Our results should be considered within the experimental conditions, facing natural limitations of comparing in vitro and in vivo studies. In vivo, the alkalizing abilities of calcium silicate-based materials can be reduced by the buffering effect of dentin. However, dentin seems to be a stronger buffer for acids than for alkalis [50]. It should also be remembered that a permanent exchange of tissue fluids at the material interface will reduce the pH level in the clinical situation [44]. Despite these facts, using calcium silicate-based materials with prolonged alkaline properties may benefit anti-inflammatory and mineralization activity. For clinicians, the setting time of MTA-like cements is considered a critical issue in clinical application. TheraCal LC, as a light-cured material, had the shortest time setting compared to all tested materials but also showed the lowest pH level. The setting reaction of the material may be one of the potential causes of changes in alkaline properties.

## 6. Limitations

Our research only evaluates the pH under specific conditions. The differences in the obtained results can be seen in the size of the samples, the lack of medium exchange resulted in the accumulation of ions, the different solubility, the water sorption, the porosity after material immersion, and perhaps the hydration process of individual materials which could impact the materials’ microstructure and ions release potential. More advanced research (for example, EDX, XRD, SEM) would confirm our assumption or give specific explanations.

## 7. Conclusions

In conclusion, the current study provides important data about the alkalizing properties of some new calcium silicate-based materials. Based on our findings, all tested materials exhibited hydroxyl ion release resulting in a rise in pH until the end of the time of experience. From all tested materials, the highest pH mean values after one-year observations showed OrthoMTA. Significant differences were also observed in different experimental groups at various periods.

## Figures and Tables

**Table 1:** Composition of calcium silicate-based materials as provided by manufacturer.

Cement (Manufacturer)	Composition
TheraCal LC (Bisco Inc., Schaumburg, IL, USA)	Light cure paste: type III Portland cement, Sr glass, fumed silica, barium sulfate, barium zirconate, and resin-containing bisphenol A-glycidyl methacrylate, urethane dimethacrylate, triethylene glycol dimethacrylate, hydroxyethyl methacrylate, and polyethylene glycol dimethacrylate
MTA Plus (PrevestDenpro, Jammu, India for Avalon Biomed Inc., Bradenton, FL, USA)	Powder: tricalcium silicate, dicalcium silicate, bismuth oxide, calcium sulphate, and silica Liquid: water or an anti-washout gel
Biodentine (Septodont, Saint- Maur-des-Fossés, France)	Powder: tricalcium silicate, dicalcium silicate, calcium carbonate, calcium oxide, and zirconium oxide as a radiopacifier Liquid: water, calcium chloride solution, and hydrosoluble polymer
RetroMTA (BioMTA, Seoul, Republic of Korea)	Powder: Tricalcium silicate, dicalcium silicate, tricalcium aluminate, tetracalciumaluminoferrite, free calcium oxide, bismuth oxide Liquid: deionized water
MTA Flow (Ultradent Products, Inc., South Jordan, UT, USA)	Powder: di- and tricalcium silicate Liquid: water-based gel
OrthoMTA (BioMTA, Seoul, Korea)	Powder: Tricalcium silicate, dicalcium silicate, tricalcium aluminate, tetracalciumaluminoferrite, free calcium oxide, bismuth oxide Liquid: deionized water

**Table 2:** pH values recorded at different time periods (mean, SD, and minimum–maximum).

Material	Time (Hour/Day/Week/Year)								
	1 H	3 H	1 D	2 D	3 D	1 W	2 W	3 W	1 Y
TheraCal	9.79 ± 0.25 (9.48–10.1) ABCE a	10.17 ± 0.10 (10.09–10.31) ABC abcdefgh	10.53 ± 0.19 (10.35–10.83) ABCDE bcdefgh	10.55 ± 0.16 (10.35–10.69) A cdefgh	10.40 ± 0.39 (9.88–10.75) A defgh	10.43 ± 0.45 (9.89–10.81) A efgh	10.57 ± 0.39 (10.07–10.88) A fgh	10.69 ± 0.34 (10.22–10.97) AB gh	10.72 ± 0.41 (10.25–11.23) A h
MTA Plus	10.28 ± 0.24 (10.01–10.48) AGHIJ ab	10.54 ± 0.30 (10.24–10.93) ADEF acd	10.98 ± 0.34 (10.68–11.45) AGHI bcefghi	11.24 ± 0.13 (11.14–11.47) BCD defghij	11.42 ± 0.09 (11.28–11.53) BCD fghij	11.51 ± 0.10 (11.34–11.59) BCDE ghij	11.59 ± 0.43 (10.83–11.87) BCDE hij	11.41 ± 0.77 (10.03–11.87) ACDEF ij	11.91 ± 0.18 (11.64–12.11) BCDE j
Biodentine	9.65 ± 0.14 (9.45–9.77) BGK ab	9.85 ± 0.11 (9.74–9.98) B acde	10.03 ± 0.35 (9.62–10.33) B bcfg	10.35 ± 0.40 (9.79–10.77) A dfh	10.51 ± 0.50 (9.85–10.93) A egh	11.11 ± 0.48 (10.36–11.67) BGHI ij	11.45 ± 0.38 (10.79–11.76) BGHI ij	11.50 ± 0.33 (10.92–11.77) BCHIJ j	12.19 ± 0.05 (12.1–12.24) BGHI k
RetroMTA	10.10 ± 0.28 (9.77–10.50) CHKLM a	10.71 ± 0.14 (10.48–10.86) DHI b	10.99 ± 0.19 (10.77–11.20) CGJK c	11.25 ± 0.16 (11.01–11.41) BFG cde	11.35 ± 0.18 (11.14–11.50) BFG defg	11.46 ± 0.18 (11.19–11.61) CGJK efg	11.59 ± 0.10 (11.47–11.69) CGJK fg	11.59 ± 0.09 (11.5–11.69) DHKL g	11.86 ± 0.05 (11.79–11.93) CGJ h
MTA Flow	10.50 ± 0.37 (10.12–10.96) DILN a	10.72 ± 0.31 (10.34–11.05) EHJ ab	11.02 ± 0.21 (10.76–11.25) DHJL bc	11.37 ± 0.10 (11.21–11.51) CFH cde	11.62 ± 0.08 (11.55–11.76) CFH defg	11.63 ± 0.12 (11.47–11.78) DHJL efg	11.91 ± 0.17 (11.61–12.04) DHJ fgh	11.91 ± 0.17 (11.63–12.08) EIKM gh	12.20 ± 0.18 (11.97–12.43) DHJK h
OrthoMTA	10.41 ± 0.51 (9.89–11.12) EJMN ab	10.52 ± 0.19 (10.34–10.85) CFIJ ac	10.81 ± 0.22 (10.54–11.11) EIKL bcd	11.20 ± 0.15 (11.01–11.4) DGH def	11.45 ± 0.19 (11.12–11.59) DGH efgh	11.63 ± 0.10 (11.52–11.78) EIKL fgh	11.75 ± 0.14 (11.61–11.95) EIKL gh	11.78 ± 0.17 (11.54–11.96) FJLM h	12.29 ± 0.27 (12.01–12.69) EIK i
Control group	6.80 ± 0.02 (6.77–6.88) F a	6.82 ± 0.10 (6.70–7.00) G a	6.84 ± 0.18 (6.60–7.10) F a	6.80 ± 0.02 (6.77–6.82) E a	6.88 ± 0.16 (6.60–7.02) E a	6.80 ± 0.12 (6.64–7.00) F a	6.82 ± 0.08 (6.70–6.90) F a	6.81 ± 0.10 (6.72–7.00) G a	6.85 ± 0.18 (6.78–7.20) F a

Different capital letters indicate significant differences (*p* < 0.05) between materials within a particular time. Different small letters indicate statistical differences (*p* < 0.05) between individual times within a material.

## Data Availability

Not applicable.
